# Postexposure Prophylaxis With rVSV-ZEBOV Following Exposure to a Patient With Ebola Virus Disease Relapse in the United Kingdom: An Operational, Safety, and Immunogenicity Report

**DOI:** 10.1093/cid/ciz1165

**Published:** 2019-11-30

**Authors:** Chris Davis, Tom Tipton, Suleman Sabir, Celia Aitken, Susan Bennett, Stephan Becker, Tom Evans, Sarah Katharina Fehling, Rory Gunson, Yper Hall, Celia Jackson, Ingolfur Johanssen, Marie Paule Kieny, Jim Mcmenamin, Elizabeth Spence, Thomas Strecker, Catie Sykes, Kate Templeton, Fiona Thorburn, Erica Peters, Ana Maria Henao Restrepo, Beth White, Maria Zambon, Miles W Carroll, Emma C Thomson

**Affiliations:** 1 Medical Research Council–University of Glasgow Centre for Virus Research, Glasgow, United Kingdom; 2 Porton Down, National Infection Service, Public Health England, Salisbury, United Kingdom; 3 West of Scotland Specialist Virology Centre, Glasgow Royal Infirmary, Glasgow, United Kingdom; 4 Institute of Virology, Philipps University Marburg, Marburg, Germany; 5 Department of Infectious Diseases, Queen Elizabeth University Hospital, Glasgow, United Kingdom; 6 Department of Laboratory Medicine, Royal Infirmary of Edinburgh, Edinburgh, United Kingdom; 7 World Health Organization, Geneva, Switzerland; 8 Inserm, Paris, France; 9 Health Protection Scotland, Glasgow, United Kingdom; 10 Public Health England Colindale, London, United Kingdom

**Keywords:** Ebola virus, rVSV-ZEBOV, vaccine, T cell

## Abstract

**Background:**

In October 2015, 65 people came into direct contact with a healthcare worker presenting with a late reactivation of Ebola virus disease (EVD) in the United Kingdom. Vaccination was offered to 45 individuals with an initial assessment of high exposure risk.

**Methods:**

Approval for rapid expanded access to the recombinant vesicular stomatitis virus–Zaire Ebola virus (rVSV-ZEBOV) vaccine as an unlicensed emergency medicine was obtained from the relevant authorities. An observational follow-up study was carried out for 1 year following vaccination.

**Results:**

Twenty-six of 45 individuals elected to receive vaccination between 10 and 11 October 2015 following written informed consent. By day 14, 39% had seroconverted, increasing to 87% by day 28 and 100% by 3 months, although these responses were not always sustained. Neutralizing antibody responses were detectable in 36% by day 14 and 73% at 12 months. Common side effects included fatigue, myalgia, headache, arthralgia, and fever. These were positively associated with glycoprotein-specific T-cell but not immunoglobulin (Ig) M or IgG antibody responses. No severe vaccine-related adverse events were reported. No one exposed to the virus became infected.

**Conclusions:**

This paper reports the use of the rVSV-ZEBOV vaccine given as an emergency intervention to individuals exposed to a patient presenting with a late reactivation of EVD. The vaccine was relatively well tolerated, but a high percentage developed a fever ≥37.5°C, necessitating urgent screening for Ebola virus, and a small number developed persistent arthralgia.

## Teaser

The rVSV-ZEBOV vaccine was used as postexposure prophylaxis in individuals exposed to Ebola virus in the United Kingdom. It was rolled out rapidly and was generally well tolerated. Side effects correlated with the magnitude of CD8+ T-cell responses.

The 2013–2016 Ebola virus (EBOV) outbreak in West Africa resulted in 28 646 reported cases of Ebola virus disease (EVD) and 11 323 deaths [1]. Healthcare workers were at particularly high risk of infection, with at least 500 deaths among 900 cases and amplified transmission of the disease in some healthcare settings. On 29 December 2014, a nurse who had worked in a treatment center in Sierra Leone was diagnosed with EVD on return to the United Kingdom [2]. Full protocols for the management of viral hemorrhagic fever were instituted immediately. Of 3 individuals providing direct healthcare to the patient prior to transfer to the UK high-level isolation unit, none were categorized as high risk due to appropriate use of personal protective equipment (PPE). In contrast, when the same patient became unwell with a previously unreported complication of EVD reactivation associated with meningo-encephalitis between 5 and 9 October 2015 (the only reported late reactivation resulting in detectable viremia of 28 646 cases), 45 healthcare workers and household contacts were initially categorized as high risk. An incident management team (IMT) was set up in order to consider postexposure prophylaxis (PEP).

In October 2015, no licensed EBOV-PEP was available, although vaccine responses had been shown to occur rapidly in macaques and humans. An interim phase III cluster-randomized trial of the replication competent recombinant vesicular stomatitis virus–Zaire Ebola virus (rVSV-ZEBOV) vaccine, published in July 2015, indicated 100% efficacy at 10 days postvaccination and an acceptable side-effect profile [3]. In rhesus macaques, it was found to provide protection when given as early as 1 week prior to exposure [4] and had also been used successfully as PEP 49 hours after exposure in a laboratory worker following a high-titer needlestick injury [5]. Another 6 individuals subsequently received the vaccine following exposure during the 2013–2016 outbreak and none developed EVD [6].

In view of the evidence of a rapid immune response in vaccinated individuals and the reported safety of the rVSV-ZEBOV vaccine, a decision was made to offer vaccination to those with the highest exposure risk. Vaccinated individuals were subsequently enrolled into the Glasgow Ebola Vaccine Follow-up Study (GEVS). Primary outcomes included evidence of infection with EBOV, the immune response following vaccination, and side effects.

## METHODS

### Approval Process

**Figure 1.**
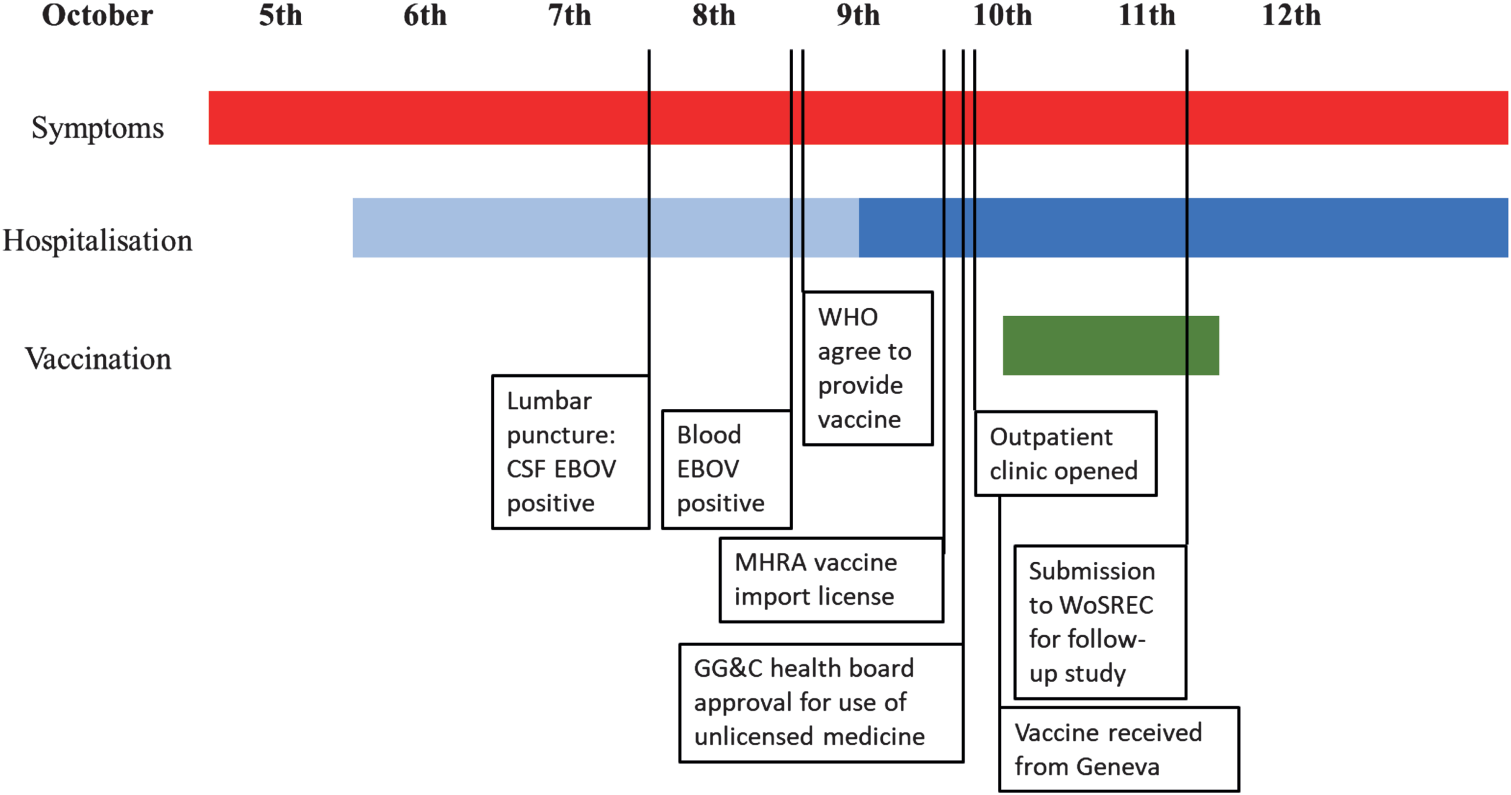
Timeline of exposure period and vaccine delivery. Following the recommendation to offer rVSV-ZEBOV vaccine by an expert panel, rapid approvals from the health board, MHRA, and local ethics committee were obtained. Abbreviations: CSF, cerebrospinal fluid; EBOV, Ebola virus; GG&C, Greater Glasgow and Clyde; MHRA, Medicines and Healthcare products Regulatory Agency; rVSV-ZEBOV, recombinant vesicular stomatitis virus–Zaire Ebola virus; WHO, World Health Organization; WoSREC, West of Scotland Research Ethics Committee.

**Figure 2.**
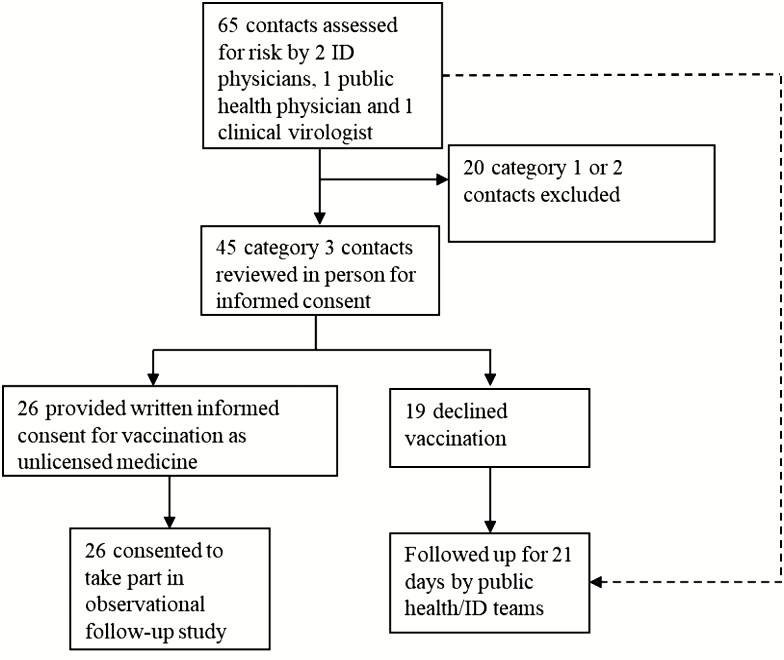
Selection of individuals for vaccination and follow-up study. Sixty-five contacts of the index patients were assessed by a team of healthcare specialists, 45 of whom were asked to attend an outpatient follow-up clinic based on exposure risk. Of these, 26 received the rVSV-ZEBOV vaccine. Abbreviations: ID, infectious diseases; rVSV-ZEBOV, recombinant vesicular stomatitis virus–Zaire Ebola virus.

An international IMT including infection experts from Europe and the United States recommended that vaccination be offered to those with the highest exposure risk on 9 October 2015, following EVD diagnosis in the index case (Figure 1). Sixty-five individuals were identified by the Greater Glasgow and Clyde public health team and designated as category 1, 2, or 3 depending on their level of exposure following national guidance (Supplementary Table 1). These cases were re-reviewed by 3 infectious diseases physicians, a public health physician, and a clinical virologist, incorporating additional expert risk categorization advice [7]. Those with a recent history of direct exposure to bodily fluids (vomit, diarrhea, blood, sweat, and/or cerebrospinal fluid [CSF]) were recalled to an emergency vaccination clinic on 10–11 October. Twenty-six of 45 clinic attendees accepted the offer of vaccination with informed consent under local National Health Service emergency regulations for unlicensed treatments (Figure 2). The following day, the West of Scotland Research Ethics Committee approved a prospective observational follow-up study (15/WS/0251).

### Vaccination

The vaccine clinic was staffed by 6 doctors, 4 nurses, and a receptionist. Any attendee with a temperature of 37.5°C or higher on arrival was immediately screened for EBOV by staff in full PPE (Tyvek suit, rubber boots, overshoes, FFP3 mask, visor, double gloves, and apron) (Supplementary Figure 1). Ebola virus polymerase chain reaction (PCR) was carried out within 6 hours of dispatch at Edinburgh Royal Infirmary. Vaccination protocols (Supplementary Information) [3] and rVSV-ZEBOV vaccine were provided by the World Health Organization (WHO) and maintained at −80 °C. A single dose of 2 × 10^7^ plaque-forming units (pfu)/mL was prepared using the PREVAIL pharmacy manual [8] and administered intramuscularly in the deltoid muscle.

### Clinical Follow-up

All individuals with a category 3 exposure were followed up with daily temperature screening for 3 weeks. Those with a temperature of 37.5°C or higher were tested for EBOV infection by reverse transcriptase (RT)–PCR. Vaccinated individuals were followed up at 30 minutes, 14 days, and 1, 3, 6, 9, and 12 months postvaccination. Twenty milliliters of blood, urine, and sputum samples and a semen sample from males were obtained at each clinic visit.

### Laboratory Methods

#### Polymerase Chain Reaction

Samples obtained during fever and at days 14 and 28 following vaccination were tested at Edinburgh Royal Infirmary using an adapted version of the Trombley PCR assay [9]. Blood, urine, and semen were tested for the presence of the rVSV-ZEBOV vaccine using a vesicular stomatitis virus (VSV) RT-PCR [10]. Equine arteritis virus was used as an internal control and had a detection limit of between 50 and 5 pfu/mL. Plasma, urine, sputum, serum, saliva, semen, and whole blood were extracted using the NucliSens EasyMAG (bioMérieux) according to the manufacturer’s instructions. Polymerase chain reaction was performed on 6 μL of RNA extract with the Platinum RT-PCR mastermix kit (Invitrogen) on an ABI Prism 7500 SDS real-time platform (Applied Biosystems) in a 15-μL reaction volume. The following thermal profile was used: 15 minutes at 50°C and then at 95°C for 15 minutes, followed by 40 cycles of 95°C for 60 seconds and 60°C for 60 seconds.

#### Immunological Assays

Antibody assays were carried out at days 14 and 28 and at 3, 6, 9, and 12 months postvaccination, and T-cell responses measured by interferon (IFN) γ ELISpot and flow cytometry at 1, 3, 6, 9, and 12 months.

### Enzyme-linked Immunosorbent Assay [11]

High-binding microtiter plates were coated with 1 µg/mL EBOV glycoprotein (GP) and incubated for 16–20 hours [11]. Following washing (phosphate-buffered saline/0.1% Tween20) and blocking (casein), 1:200 dilutions of plasma sample were added and incubated for 2 hours. Polyclonal antihuman immunoglobulin (Ig) G–AP antibody (1:1000) with substrate (diethanolamine substrate buffer with 20 mg p-Nitrophenyl Phosphate in 20 mL double distilled water) was used to develop the reaction. Optical density was determined at 405 nm. Samples were analyzed in duplicate, and background was subtracted from the mean of each sample. Plates were read using a predefined softmax template, which fits a 4-parameter logistic model to the dose-response data; IU/mL are based on the WHO International Reference Standard (NIBSC 15/220), which was used to quantify the internal standard.

#### Neutralization Assays

Neutralization assays were performed at biosafety level 4 at the Institute of Virology, Philipps University Marburg, Germany, as previously described [12]. Volunteer blood plasma was incubated at 56°C for 30 minutes for complement inactivation. After centrifugation at 13 000 rpm for 10 minutes, sera were serially diluted from 2^3^–2^10^in Dulbecco’s modified Eagle’s medium (DMEM; Gibco) supplemented with 2% fetal calf serum (FCS; Gibco), penicillin (100 U/mL), streptomycin (100 mg/mL), and l-glutamine (2 mmol/L) (Invitrogen) in 96-well culture plates. One hundred Median Tissue Culture Infectious Dose (TCID50) units of EBOV (Zaire, isolate Mayinga, AF086833) were added to the serum dilutions. Following incubation at 37°C for 1 hour, Vero cell suspension in DMEM containing 2% FCS was added and incubated at 37°C with 5% CO_2_. Cytopathic effects were evaluated at 7 days postinfection. Neutralization titers were calculated as geometric mean titer of 4 replicates.

#### IFNγ-ELISpot Assays

Peripheral blood mononuclear cells (PBMCs) were thawed using warm media and rested overnight at 37°C. The following day they were stimulated with overlapping EBOV GP peptides (MP1/MP2), as previously described [13]. Plates were counted using an S6 core analyzer (Cellular Technology Limited), and results adjusted to spot-forming units/1 × 10^6^ cells/mL. Analysis required detection of a positive control, then subtraction of the non–peptide-stimulated control from peptide-stimulated samples.

#### T-cell Phenotyping Studies

Intracellular cytokine staining was performed as previously described [13]. Briefly, PBMCs were resuspended in warmed media and rested overnight at 37°C. The following day, cells were adjusted to 1 × 10^6^ cells/mL in media containing anti-CD28, CD49d, and CD107a-PerCP cy5.5 (1 µg/mL). Cells were then untreated or stimulated with EBOV GP peptide pool, containing 187 × 15-mer overlapping peptides at 2.5 µg/peptide or 1 µg/mL Staphylococcal Enterotoxin B peptide for 16–18 hours. After 2 hours, brefeldin A and monensin (1 µg/mL) were added to block cytokine secretion. The following day, samples were washed and LIVE/DEAD dye added. Samples were washed, incubated with cell surface antibodies (CD3-APC 750, CD4-BV786, CD8-AF700, CD19-BV510, and then CD14-BV510, CCR7-APC, CD95-BV395, and CD45RO-BV605); then washed, fixed, and permeabilized; then stained for intracellular cytokines using IFNγ-AF488, TNFα-BV421, and IL-2-PE. Samples were analyzed using a BD Fortessa machine and FACS Diva, FlowJo, Pestle, and SPICE software (see Supplementary Information).

### Statistical Analysis

Comparisons were made using parametric or nonparametric methods, as appropriate, using STATA version 10 (StataCorp).

## RESULTS

Of 65 individuals designated as having had contact with the infected patient, 45 category 3 contacts were found to have had possible direct skin contact with contaminated bodily fluids (vomit, sweat, blood, urine, or CSF). None had evidence of percutaneous exposure and all would be categorized as “intermediate” in a more recently proposed exposure risk stratification [7]. Of these, 26 elected to receive vaccination following written informed consent and agreed to be followed as part of the observational GEVS. The median age of those vaccinated was 40 years (range, 24–67 years). Fifteen of 26 (58%) were healthcare workers and 11 of 26 (42%) were household contacts. All individuals were followed up within the first 3 months following vaccination, but attendance at subsequent follow-up clinics was incomplete due to movement of medical and nursing staff to other cities within the United Kingdom (Supplementary Table 2). No one exposed to the virus became infected. All samples tested for EBOV and VSV were negative, including 2 febrile clinic attendees tested for EBOV prior to vaccination.

### Antibody Responses

**Figure 3.**
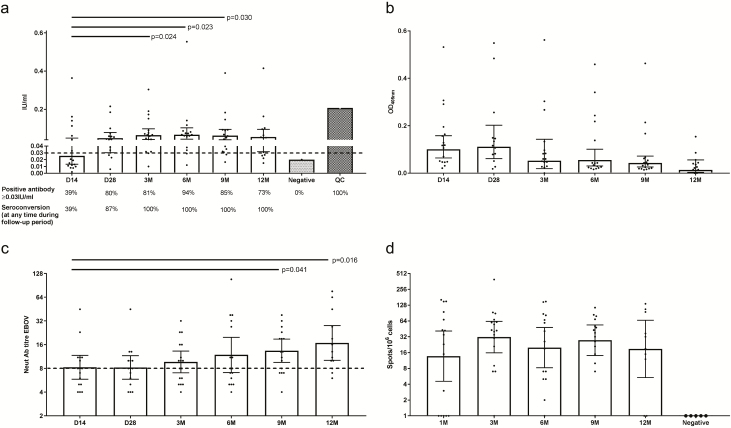
Adaptive immune responses over time in vaccinated individuals. Antibody responses measured by indirect ELISA, IgM, neutralization, and IFNγ ELISpot are plotted over time. Anti-GP seroconversion rates are shown with a cutoff of 0.03 IU/mL (3 × standard deviation of negative control). For antibody assays, negative control levels from unvaccinated individuals are plotted with a positive QC (dotted bars). Bars show the geometric mean with 95% confidence interval. Numbers below the bars show the percentage of seroconverted people. Each dot is the average from 2 separate assays. For ELISpot, negative control represents unstimulated cells from vaccinated patients. *P* values < .05 (Mann-Whitney *U* test) are highlighted. *A*, Total anti-GP responses over time measured by indirect ELISA. *B*, Anti-GP IgM responses by ELISA over time. *C*, Neutralization responses over time. *D*, T-cell responses by IFNγ ELISpot to EBOV GP peptides over time. Abbreviations: D, day; EBOV, Ebola virus; ELISA, enzyme-linked immunosorbent assay; GP, glycoprotein; IFNγ, interferon γ; IgM, immunoglobulin M; M, months; Neut Ab, neutralizing antibodies; OD, optical density; QC, quality control.

IgG indirect enzyme-linked immunosorbent assay (ELISA) results are presented in Figure 3A as seroconversion (a positive antibody response at any time during the follow-up period) and individual responses detected at each follow-up visit to assess longevity of the response. By day 14, 39% had seroconverted. This increased to 87% by day 28 and 100% by 3 months. Such responses were not always sustained; one 68-year-old individual developed a positive IgG response at 14 days postvaccination, but the level descended below the detection threshold at all further time points (Supplementary Figure 2). This was not associated with the onset of any form of illness or immunosuppressive treatment during this time period. Detectable antibody responses fell to 73% by 12 months postvaccination. Individual results are shown in Supplementary Figure 3. A positive anti-GP IgM response peaked at 14–28 days postvaccination (Figure 3B) and negatively correlated with the emergence of neutralizing antibody responses (Supplementary Figure 4). Neutralizing antibody responses were detectable in 6 of 16 (36%) individuals by day 14 and peaked at 9–12 months postvaccination, with a detectable response in 9 of 12 individuals (73%) (Figure 3C).

### T-cell Responses

IFNγ ELISpot responses to GP were detected at all time points, followed a similar pattern to neutralization over time (Supplementary Figure 5), and peaked at 6 months postvaccination (Figure 3D). Individual responses are shown in Supplementary Figure 6.

### Side Effects

Side effects were common but mild in the majority of cases and were characterized by a syndrome of fatigue, myalgia, headache, and arthralgia (Tables 1 and 2). The presence of 1 symptom was strongly associated with the presence of others (Fisher’s exact test, *P* < .0001). During the first 72 hours of follow-up, 50% of individuals developed a fever of 37.5°C or higher, requiring in-hospital assessment and testing for EBOV. While the median duration of side effects was 0–1 days, a small number of patients developed long-standing symptoms of fatigue (up to 343 days), arthralgia (up to 261 days), and headache (up to 108 days). Two patients experienced long-lasting symptoms of arthralgia, one of whom had a diagnosis of osteoarthritis and flexor tendonitis thought to be unrelated to vaccination following specialist rheumatological review. Further details on cases of arthralgia are shown in Supplementary Table 3.

**Table 1.** Side Effects Associated With rVSV-ZEBOV Vaccination Specified in the Follow-up Questionnaire

Side Effect	Percentage (n/N)	Duration (IQR), days	Severity (1–5) (IQR), days	Details
Fatigue	81 (21/26)	2 (1–5)	1 (1–2.5)	…
Pain at injection site	69 (18/26)	2 (1–4)	1 (1–2)	…
Myalgia	69 (18/26)	2.5 (1–4.5)	1 (1–2)	…
Headache	69 (18/26)	2 (1–4)	1 (1–2)	38% reported migraines
Arthralgia	54 (14/26)	2.5 (1–17.75)	2 (1–2.75)	Two patients with long-lasting symptoms
Fever (≥37.5°C)	50 (13/26)	…	…	All tested negative for EBOV
Diarrhea	15 (4/26)	1 (1–1)	1 (1–1)	…
Vomiting	8 (2/26)	18.5 (1–36)	2.5 (1–4)	…
Induration	0 (0/26)	0 (0–0)	0 (0–0)	…

Abbreviations: EBOV, Ebola virus; IQR, interquartile range; rVSV-ZEBOV, recombinant vesicular stomatitis virus–Zaire Ebola virus.

**Table 2.** Side Effects Associated With rVSV-ZEBOV Vaccination Volunteered During Follow-up

Other Reported Side Effects	Percentage (n/N)	Related to Vaccine
Ophthalmic shingles	3 (1/26)	Possible
Fractured neck of femur	3 (1/26)	Unrelated
Dizziness	6 (2/26)	Likely
Sinusitis	3 (1/26)	Unrelated
Cervical lymphadenopathy	3 (1/26)	Related (evaluated by ultrasound and too small for aspiration)

Abbreviation: rVSV-ZEBOV, recombinant vesicular stomatitis virus–Zaire Ebola virus.

**Figure 4.**
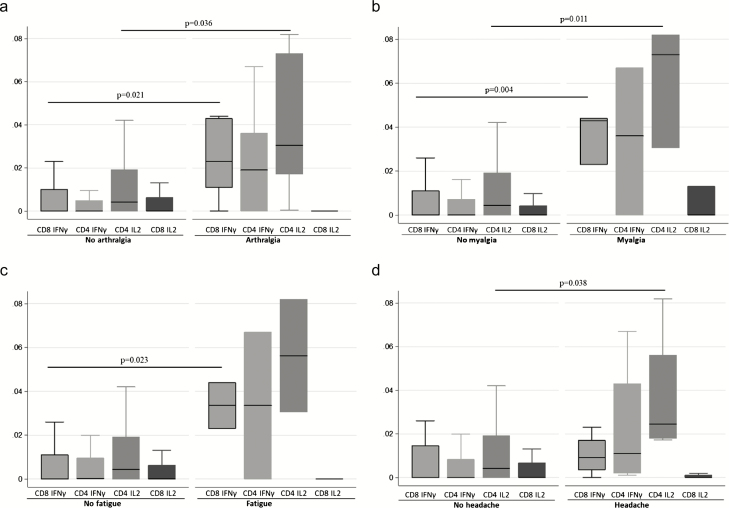
Side effects related to T-cell response. CD4+ and CD8+ IFNγ and CD4+ IL2 responses in individuals with arthralgia (*A*), myalgia (*B*), fatigue (*C*), and headache (*D*) are shown. *P* values <.05 (Mann-Whitney *U* test) are highlighted. Abbreviations: IFNγ, interferon γ; IL2, interleukin 2.

Symptoms of arthralgia, myalgia, and fatigue occurring at the time of sampling were significantly associated with a higher proportion of CD8+ IFNγ and CD4+ interleukin (IL) 2–secreting cells, while headache was associated with higher CD4+ IL2 (Figure 4) and IFNγ ELISpot response. No significant association with IgM, IgG, or neutralizing antibody responses was found (Supplementary Table 4).

## DISCUSSION

The risk of transmission of EBOV to household contacts and healthcare workers exposed to infected bodily fluids is high, particularly prior to diagnosis when the risks may not be fully appreciated. During the West Africa 2013–2016 outbreak, several infected individuals travelled by air to other countries, resulting in onward transmission. In Spain, a nurse became infected after caring for a patient transferred for specialist care, and in the United States, 2 nurses became infected after contact with an undiagnosed infected traveller. In Nigeria, 20 people were infected (11 healthcare workers) following a single introduction [14]. No randomized studies on the use of PEP have been carried out in humans, but vaccination and antiviral agents have been studied in exposed individuals on a case-by-case basis [6, 7] and more recently in a large outbreak in the Democratic Republic of Congo.

The rVSV-ZEBOV vaccine is a highly effective vaccine that rapidly protects mice, hamsters, guinea pigs, nonhuman primates, and humans from infection with EBOV when administered prior to exposure. In humans, ring vaccination with rVSV-ZEBOV at a dose of 2 × 10^7^ pfu was highly effective at preventing infection in contacts and contacts of contacts of individuals with EBOV infection in West Africa in a large phase III trial [15]. In this study, which initially involved an immediate and a delayed vaccine arm, no infections occurred 10 days after vaccination in any recipient (100% vaccine efficacy). As a result, randomization was halted by an independent safety board and all subsequent participants in the study were offered immediate vaccination. Vaccination was carried out a median of 7.3 and 9.8 days following index patient symptom onset in the immediate and nonrandomized vaccine rings, respectively. Importantly, EBOV infection did occur in the 10-day period postvaccination, and this was not reduced compared with the delayed vaccination arm. This indicates that the timing of the use of the vaccine is likely to be critical and would need to stimulate a protective immune response early within the median 9- to 10-day incubation period.

In rhesus macaques (in which infection is uniformly fatal with a more rapid onset of disease [6]), a single dose of the vaccine provides complete protection when given as few as 7 days before challenge [4, 16] and prevents infection in 50% when given as PEP 24 hours after infection [17]. Immunity is likely to be largely innate or antibody dependent as depletion of CD4 or CD8+ cells postchallenge does not abrogate protection [18].

The first use of rVSV-ZEBOV in a human was reported in 2011 following a high-titer needlestick exposure in a laboratory [5]. In this case, a single dose of 5 × 10^7^ pfu was administered 48 hours after the accident. At least 6 other individuals have now also received the vaccine, given 1–3 days postexposure, with the majority having been exposed in Ebola treatment units during the 2014–2016 West Africa outbreak [5, 19, 20]. All of these individuals were given a higher dose of vaccine and all developed significant side effects, although none became infected (Table 3).

**Table 3.** Case Reports/Series of Individuals Treated With rVSV-ZEBOV Vaccine

Reference	Exposure Type	Setting	Timing of Vaccination	Dose of Vaccine	Outcome
Günther et al (2011) [5]	High-risk needlestick injury	Laboratory (BSL4)	48 hours	5 × 10^7^ pfu	Uninfected, symptomatic
Wong et al (2016) [19]	High-risk injury with glass	Clinical (ETU)	3 days	1 × 10^8^ pfu	Uninfected, symptomatic
Wong et al (2016) [19]	Low-risk needlestick injury	Clinical (ETU)	24 hours	1 × 10^8^ pfu	Uninfected, symptomatic
Wong et al (2016) [19]	High-risk injury with glass	Clinical (ETU)	27 hours	1 × 10^8^ pfu	Uninfected, symptomatic
Wong et al (2016); Lai et al (2015)^a^ [19, 29]	High-risk needlestick injury	Clinical (ETU)	43 hours	1 × 10^8^ pfu	Uninfected, symptomatic
Wong et al (2016) [19]	High-risk needlestick injury	Clinical (ETU)	3 days	1 × 10^8^ pfu	Uninfected, symptomatic
Cnops et al (2015) [20]	High-risk needlestick injury	Clinical (ETU)	2 days	1 × 10^8^ pfu	Uninfected, symptomatic

Abbreviations: BSL4, bio-safety level 4; ETU, Ebola treatment unit; pfu, plaque-forming units; rVSV-ZEBOV, recombinant vesicular stomatitis virus–Zaire Ebola virus.

^a^Same individual reported in 2 separate publications.

In this intervention, the 2 × 10^7^–pfu dose was selected as a balance between very high levels of reactogenicity found with the 1 × 10^8^–pfu dose and the lower immune responses found in individuals treated with lower doses in phase I and II studies [10, 12, 21]. We detected a higher incidence of symptoms in our study compared with these trials [10] but lower than that found in the cases described in Table 3. The high incidence of symptoms may be related to variation in genetic background and high levels of psychological stress.

The risk of infection was likely to have been highest in those who had contact with body fluids from the index case. While blood and CSF tested positive by PCR, infectious virus was only isolated from the CSF where the titer was highest [2] (vomit, urine, saliva, and rectal swabs all subsequently tested PCR negative). In retrospect, the individuals with the highest potential risk of transmission were those exposed to CSF during the lumbar puncture procedure that took place 3 days before vaccination.

The mechanism of protection following vaccination with rVSV-ZEBOV may involve innate, T-cell–mediated, and/or B-cell–mediated responses [15, 22, 23]. We assessed the immune response by indirect ELISA, neutralization with live ZEBOV (Mayinga strain), ELISpot, and flow cytometry. There are no definite surrogates of immunity, but such responses have been associated with protection from infection in macaques and humans. IgM responses peaked at day 14, while IgG seroconversion occurred in 39% at 14 days postvaccination, increasing to 87% by day 28 and to 100% of individuals by 3 months. The day 14 anti-GP seroconversion was lower than that found in rhesus macaques [4, 24, 25] and in human participants in pooled North American phase I studies, which showed universal seroconversion by day 14 [26]. In the phase II PREVAIL trials, 77–83% of 500 individuals seroconverted within 1 month of vaccination [8, 27]. We found that the numbers of individuals with positive neutralizing antibody responses were similar to those with anti-GP responses evaluated by ELISA. Seventy-five percent of individuals were anti–GP positive at 1 year after vaccination and 73% had positive neutralization results. This is in keeping with a long-lasting effect found in other studies [28]. T-cell responses directed against GP were also detected, as described previously [23].

Future use of rVSV-ZEBOV vaccine must be balanced against the risk of side effects. It is a live vaccine, and fever in vaccinated individuals has been found to be associated with evidence of replicating rVSV-ZEBOV in blood [29]. The side-effect profile of these Scottish vaccine recipients was similar to recipients in Switzerland with a higher prevalence of arthralgia than reported in phase I studies in Germany and Kenya. Arthralgia in Swiss participants lasted a median of 8 days (range, 3–167 days; interquartile range, 4–87 days) [12]. As in this study, symptoms were generally short lived but were longer lasting in 2 patients. Headache, fatigue, myalgia, and arthralgia were associated with the magnitude of T-cell response to pooled GP peptides, with higher CD4+ production of IL2 and CD8+ production of IFNγ, but not with antibody responses.

There were several limitations to this study. First, we cannot comment on efficacy of the vaccine as this was not a randomized controlled intervention following definitive virus exposure. However, we have demonstrated that Ebola vaccine used as PEP was immunogenic and relatively well tolerated. Timing of administration is likely to be critical as some individuals did not develop a rapid immune response. While vaccination is a reasonable PEP strategy, other interventions such as the use of antiviral agents or newer vaccines may be warranted.

## Supplementary Data

Supplementary materials are available at *Clinical Infectious Diseases* online. Consisting of data provided by the authors to benefit the reader, the posted materials are not copyedited and are the sole responsibility of the authors, so questions or comments should be addressed to the corresponding author.

ciz1165_suppl_Supplementary_Table

ciz1165_suppl_Supplementary_Forms

ciz1165_suppl_Supplementary_Questionaire

## Notes

***Acknowledgments***. The rVSV-ZEBOV vaccine was provided by Merck and WHO. The authors thank the reception, nursing, and medical staff for voluntary assistance at Gartnavel General Hospital and Dr Emma Aarons, Public Health England Porton Down, for helpful advice.

***Disclaimer.*** The sponsor of the study had no role in study design, data collection, analysis or writing of the report.

***Financial support.*** This work was supported by the US Food and Drug Administration (grant number HHSF223201510104C), the German Research Foundation (grant number 197785619/SFB 1021), the Medical Research Council (grant number MC_UU_12014/1), and the Wellcome Trust (grant number 102789/Z/13/A). Vaccine was provided free of charge by Merck/WHO.

***Potential conflicts of interest.*** E. C. T. and C. D. report grants from the Wellcome Trust and the Medical Research Council during the conduct of the study. M. W. C., Y. H., and T. T. report a grant from the US Food and Drug Administration during the conduct of the study. All other authors report no potential conflicts. All authors have submitted the ICMJE Form for Disclosure of Potential Conflicts of Interest. Conflicts that the editors consider relevant to the content of the manuscript have been disclosed.
