# Supplementary material for: Postexposure Prophylaxis With rVSV-ZEBOV Following Exposure to a Patient With Ebola Virus Disease Relapse in the United Kingdom: An Operational, Safety, and Immunogenicity Report
Source: Clin Infect Dis. 2019 Nov 30;71(11):2872–9. doi: 10.1093/cid/ciz1165 (PMC7778350; doi:10.1093/cid/ciz1165)
Supplement: ciz1165_suppl_Supplementary_Table [file ciz1165_suppl_supplementary_table.docx]

**Supplementary Table 1:** **Public health recommendations for asymptomatic contacts of an Ebola case in the UK (Public Health England)**

- **Category 3** - Anyone not wearing full PPE who has had direct contact with an Ebola case body fluids
- **Category 2** - Anyone wearing full PPE who has had direct contact with an Ebola case body fluids
- **Category 1** - Encounter with the case but no direct contact with their body fluids or

other potentially infectious material

**Supplementary Table 2: Follow-up clinic attendance**

Clinic attendance is shown in black, telephone contact in grey

| **Study no.** | **7d** | **14d** | **1m** | **3m** | **6m** | **9m** | **12m** |
| --- | --- | --- | --- | --- | --- | --- | --- |
| **GEVS1** |  |  |  |  |  |  |  |
| **GEVS2** |  |  |  |  |  |  |  |
| **GEVS3** |  |  |  |  |  |  |  |
| **GEVS4** |  |  |  |  |  |  |  |
| **GEVS7** |  |  |  |  |  |  |  |
| **GEVS8** |  |  |  |  |  |  |  |
| **GEVS10** |  |  |  |  |  |  |  |
| **GEVS11** |  |  |  |  |  |  |  |
| **GEVS12** |  |  |  |  |  |  |  |
| **GEVS13** |  |  |  |  |  |  |  |
| **GEVS14** |  |  |  |  |  |  |  |
| **GEVS15** |  |  |  |  |  |  |  |
| **GEVS16** |  |  |  |  |  |  |  |
| **GEVS17** |  |  |  |  |  |  |  |
| **GEVS18** |  |  |  |  |  |  |  |
| **GEVS19** |  |  |  |  |  |  |  |
| **GEVS20** |  |  |  |  |  |  |  |
| **GEVS21** |  |  |  |  |  |  |  |
| **GEVS22** |  |  |  |  |  |  |  |
| **GEVS23** |  |  |  |  |  |  |  |
| **GEVS24** |  |  |  |  |  |  |  |
| **GEVS25** |  |  |  |  |  |  |  |
| **GEVS26** |  |  |  |  |  |  |  |
| **GEVS27** |  |  |  |  |  |  |  |
| **GEVS28** |  |  |  |  |  |  |  |
| **GEVS29** |  |  |  |  |  |  |  |

**Supplementary Table 3: Clinical details of patients reporting arthralgia**

| **Case** | **Onset post-vaccination** | **Duration** | **Number of joints** | **Which joints** | **Further detail** |
| --- | --- | --- | --- | --- | --- |
| **GEVS 1** | **1 day** | **3 days** | **3** | **Neck, knees** |  |
| **GEVS3** | **1 day** | **1 day** | **2** | **Elbows** |  |
| **GEVS4** | **1 day** | **4 days** | **Multiple** | **Wrists, knees, ribs, neck, spine** |  |
| **GEVS10** | **20 days** | **35 days** | **2** | **Right hand, carpal tunnel syndrome, left hip** |  |
| **GEVS12** | **At day 1 (hips) and 3 months (ankle)** | **1 day** | **3** | **Hips**  **Ankle – likely unrelated (running injury)** |  |
| **GEVS13** | **4 days** | **4 days** | **1** | **Lower spine** |  |
| **GEVS16** |  | **261 days** | **Multiple** | **Fingers, knee with swelling, wrists, toes, hips, knees** | **Intermittent symptoms** |
| **GEVS18** | **2 days** | **3 days** | **4** | **Wrists, shoulders** |  |
| **GEVS22** | **1 day** | **177 days** | **Multiple** | **Elbows, wrists, knees, fingers** | **Gradual resolution over time** |
| **GEVS24** | **2 days** | **2 days** | **3** | **Neck, shoulders** |  |
| **GEVS27** | **2 days** | **2 days** | **Multiple** | **Generalised** |  |
| **GEVS28** | **2 days** | **14 days** | **Multiple** | **Fingers, toes** |  |
| **GEVS29** | **2 days** | **2 days** | **1** | **Lower spine** |  |

**Supplementary Table 4: Antibody and T cell responses relationship with symptoms.** Samples were analysed using a BD Fortessa machine and FACS Diva, FlowJo™, Pestle and SPICE software^[[1]](#footnote-1),^^[[2]](#footnote-2)^.

| Side effect | T cell type | Cytokine | Peptides | p_value |
| --- | --- | --- | --- | --- |
| Arthralgia | **CD107** | **NA** | **GP** | 0.8796 |
| Arthralgia | **CD4** | **IFN** | **GP** | 0.1506 |
| Arthralgia | **CD4** | **IL2** | **GP** | **0.0364** |
| Arthralgia | **CD8** | **IFN** | **GP** | **0.0206** |
| Arthralgia | **CD8** | **IL2** | **GP** | 0.7962 |
| Arthralgia | **ELISA** | **NA** | **NA** | 0.626 |
| Arthralgia | **ELISPOT** | **NA** | **NA** | 0.3178 |
| Arthralgia | **LOGELISA** | **NA** | **NA** | 0.626 |
| Arthralgia | **CD107** | **NA** | **NP** | 0.4601 |
| Arthralgia | **CD107** | **NA** | **NP** | 0.7148 |
| Arthralgia | **CD4** | **IFN** | **NP** | 0.514 |
| Arthralgia | **CD4** | **IL2** | **NP** | 0.8882 |
| Arthralgia | **CD8** | **IL2** | **NP** | 0.8813 |
| Arthralgia | **CD8** | **IFN** | **NP** | 0.8608 |
| Arthralgia | **CD107** | **NA** | **SEB** | 0.8551 |
| Arthralgia | **CD4** | **IL2** | **SEB** | 0.6574 |
| Arthralgia | **CD4** | **IL2** | **SEB** | 0.6574 |
| Arthralgia | **CD8** | **IFN** | **SEB** | 0.2225 |
| Arthralgia | **CD8** | **IL2** | **SEB** | 0.5472 |
| Arthralgia | **CD8** | **IFN** | **SEB** | 0.5902 |
| Fatigue | **CD107** | **NA** | **GP** | 0.8926 |
| Fatigue | **CD4** | **IFN** | **GP** | 0.3622 |
| Fatigue | **CD4** | **IL2** | **GP** | **0.0568** |
| Fatigue | **CD8** | **IFN** | **GP** | **0.0303** |
| Fatigue | **CD8** | **IL2** | **GP** | 0.7346 |
| Fatigue | **ELISA** | **NA** | **NA** | 0.2825 |
| Fatigue | **ELISPOT** | **NA** | **NA** | 0.2164 |
| Fatigue | **LOGELISA** | **NA** | **NA** | 0.2825 |
| Fatigue | **CD107** | **NA** | **NP** | 0.169 |
| Fatigue | **CD4** | **IL2** | **NP** | 0.0824 |
| Fatigue | **CD4** | **IFN** | **NP** | 0.8411 |
| Fatigue | **CD8** | **IL2** | **NP** | 0.6547 |
| Fatigue | **CD8** | **IFN** | **NP** | 0.6376 |
| Fatigue | **CD107** | **NA** | **SEB** | 0.6467 |
| Fatigue | **CD4** | **IFN** | **SEB** | 0.739 |
| Fatigue | **CD4** | **IL2** | **SEB** | 0.2534 |
| Fatigue | **CD8** | **IFN** | **SEB** | 0.4049 |
| Fatigue | **CD8** | **IL2** | **SEB** | 0.6684 |
| Headache | **CD107** | **NA** | **GP** | 0.307 |
| Headache | **CD4** | **IL2** | **GP** | **0.0426** |
| Headache | **CD4** | **IFN** | **GP** | 0.0666 |
| Headache | **CD8** | **IFN** | **GP** | 0.2948 |
| Headache | **CD8** | **IL2** | **GP** | 0.9582 |
| Headache | **ELISA** | **NA** | **NA** | 0.6223 |
| Headache | **ELISPOT** | **NA** | **NA** | **0.0239** |
| Headache | **LOGELISA** | **NA** | **NA** | 0.6223 |
| Headache | **CD107** | **NA** | **NP** | **0.0473** |
| Headache | **CD4** | **IFN** | **NP** | 0.193 |
| Headache | **CD4** | **IL2** | **NP** | 0.973 |
| Headache | **CD8** | **IFN** | **NP** | 0.3261 |
| Headache | **CD8** | **IL2** | **NP** | 0.6801 |
| Headache | **CD107** | **NA** | **SEB** | 0.3056 |
| Headache | **CD4** | **IFN** | **SEB** | 0.4295 |
| Headache | **CD4** | **IL2** | **SEB** | 0.4097 |
| Headache | **CD8** | **IL2** | **SEB** | 0.3028 |
| Headache | **CD8** | **IFN** | **SEB** | 0.1181 |
| Myalgia | **CD107** | **NA** | **GP** | 0.6563 |
| Myalgia | **CD4** | **IL2** | **GP** | **0.0126** |
| Myalgia | **CD4** | **IFN** | **GP** | 0.1007 |
| Myalgia | **CD8** | **IFN** | **GP** | **0.0064** |
| Myalgia | **CD8** | **IL2** | **GP** | 0.5897 |
| Myalgia | **ELISA** | **NA** | **NA** | 0.0901 |
| Myalgia | **ELISPOT** | **NA** | **NA** | 0.0609 |
| Myalgia | **LOGELISA** | **NA** | **NA** | 0.0901 |
| Myalgia | **CD107** | **NA** | **NP** | 0.7768 |
| Myalgia | **CD4** | **IFN** | **NP** | 0.2557 |
| Myalgia | **CD4** | **IL2** | **NP** | 0.5614 |
| Myalgia | **CD8** | **IL2** | **NP** | 0.6521 |
| Myalgia | **CD8** | **IFN** | **NP** | 0.6196 |
| Myalgia | **CD107** | **NA** | **SEB** | 0.265 |
| Myalgia | **CD4** | **IL2** | **SEB** | 0.2982 |
| Myalgia | **CD4** | **IFN** | **SEB** | 0.9218 |
| Myalgia | **CD8** | **IL2** | **SEB** | 0.2982 |
| Myalgia | **CD8** | **IFN** | **SEB** | 0.7239 |
| Pain | **CD107** | **NA** | **GP** | 0.0888 |
| Pain | **CD4** | **IFN** | **GP** | 0.1686 |
| Pain | **CD4** | **IL2** | **GP** | 0.1335 |
| Pain | **CD8** | **IFN** | **GP** | 0.5708 |
| Pain | **CD8** | **IL2** | **GP** | 0.0899 |
| Pain | **ELISA** | **NA** | **NA** | 0.5227 |
| Pain | **ELISPOT** | **NA** | **NA** | 0.5567 |
| Pain | **LOGELISA** | **NA** | **NA** | 0.5227 |
| Pain | **CD4** | **IL2** | **NP** | 0.1002 |
| Pain | **CD4** | **IFN** | **NP** | 0.0736 |
| Pain | **CD8** | **IFN** | **NP** | 0.0726 |
| Pain | **CD8** | **IL2** | **NP** | 0.0818 |
| Pain | **CD107** | **NA** | **SEB** | 0.1881 |
| Pain | **CD4** | **IL2** | **SEB** | 0.5711 |
| Pain | **CD4** | **IFN** | **SEB** | 0.8156 |
| Pain | **CD8** | **IL2** | **SEB** | 0.1519 |
| Pain | **CD8** | **IFN** | **SEB** | 0.2176 |
| Temp | **ELISA** | **NA** | **NA** | 0.2023 |
| Temp | **LOGELISA** | **NA** | **NA** | 0.2023 |
| Vomiting | **CD107** | **NA** | **GP** | 0.85 |
| Vomiting | **CD4** | **IFN** | **GP** | 0.2979 |
| Vomiting | **CD4** | **IL2** | **GP** | 0.6647 |
| Vomiting | **CD8** | **IFN** | **GP** | 0.5708 |
| Vomiting | **CD8** | **IL2** | **GP** | 0.8123 |
| Vomiting | **ELISA** | **NA** | **NA** | 0.7545 |
| Vomiting | **ELISPOT** | **NA** | **NA** | 0.2276 |
| Vomiting | **LOGELISA** | **NA** | **NA** | 0.7545 |
| Vomiting | **CD107** | **NA** | **NP** | 0.7239 |
| Vomiting | **CD4** | **IFN** | **NP** | 0.3805 |
| Vomiting | **CD4** | **IL2** | **NP** | 0.3573 |
| Vomiting | **CD8** | **IFN** | **NP** | 0.7416 |
| Vomiting | **CD8** | **IL2** | **NP** | 0.7541 |
| Vomiting | **CD107** | **NA** | **SEB** | 0.2612 |
| Vomiting | **CD4** | **IFN** | **SEB** | 0.1938 |
| Vomiting | **CD4** | **IL2** | **SEB** | 0.1719 |
| Vomiting | **CD8** | **IFN** | **SEB** | 0.1719 |
| Vomiting | **CD8** | **IL2** | **SEB** | 0.2176 |

**Supplementary Figure 1: Vaccination outpatient clinic layout** The vaccine clinic was opened out of hours and a one-way system set up to minimise contact between exposed individuals. A temperature screening station was set up on entry and febrile individuals (n=2) were diverted for immediate screening for EBOV.


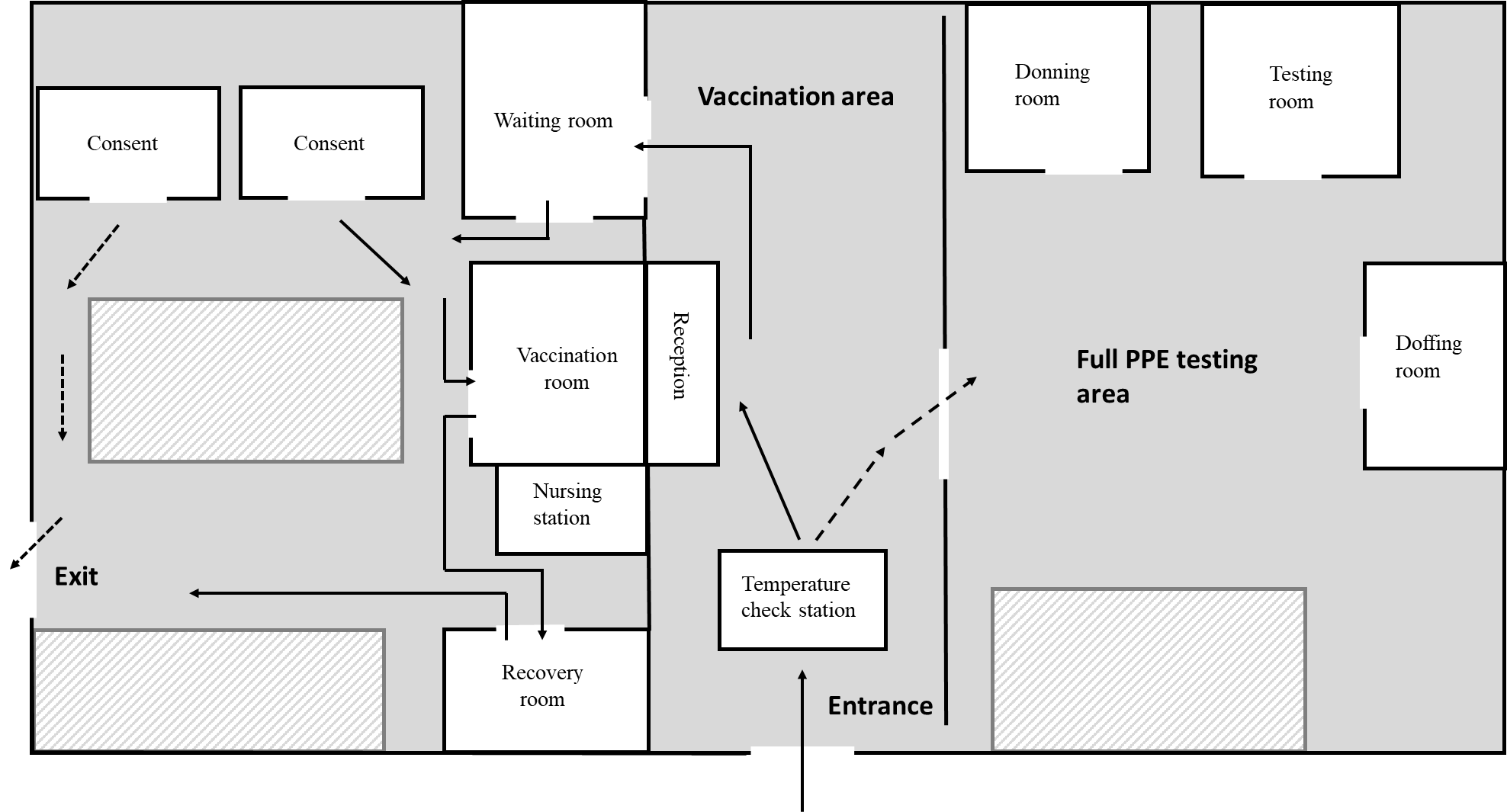


**Supplementary Figure 2: IgG indirect ELISA responses:** selected individual results are highlighted at different timepoints

**Supplementary figure 3: Ab responses:** Antibody responses assessed by ELISA and neutralisation are shown by individual over time


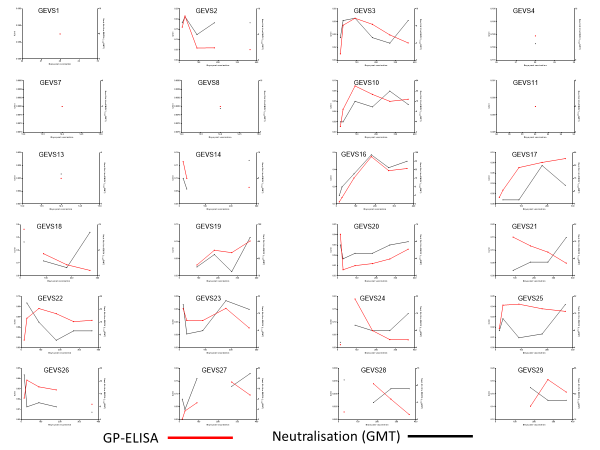


**Supplementary Figure 4: Mean IgM and neutralising Ab responses over time:** average IgM and neutralising antibody responses are plotted over time

**Supplementary Figure 5: GP ELISpot versus neutralisation:** average ELISpot and neutralising antibody responses are plotted over time

**Supplementary Figure 6: ELISpot results:** individual IFNγ responses are plotted over time*
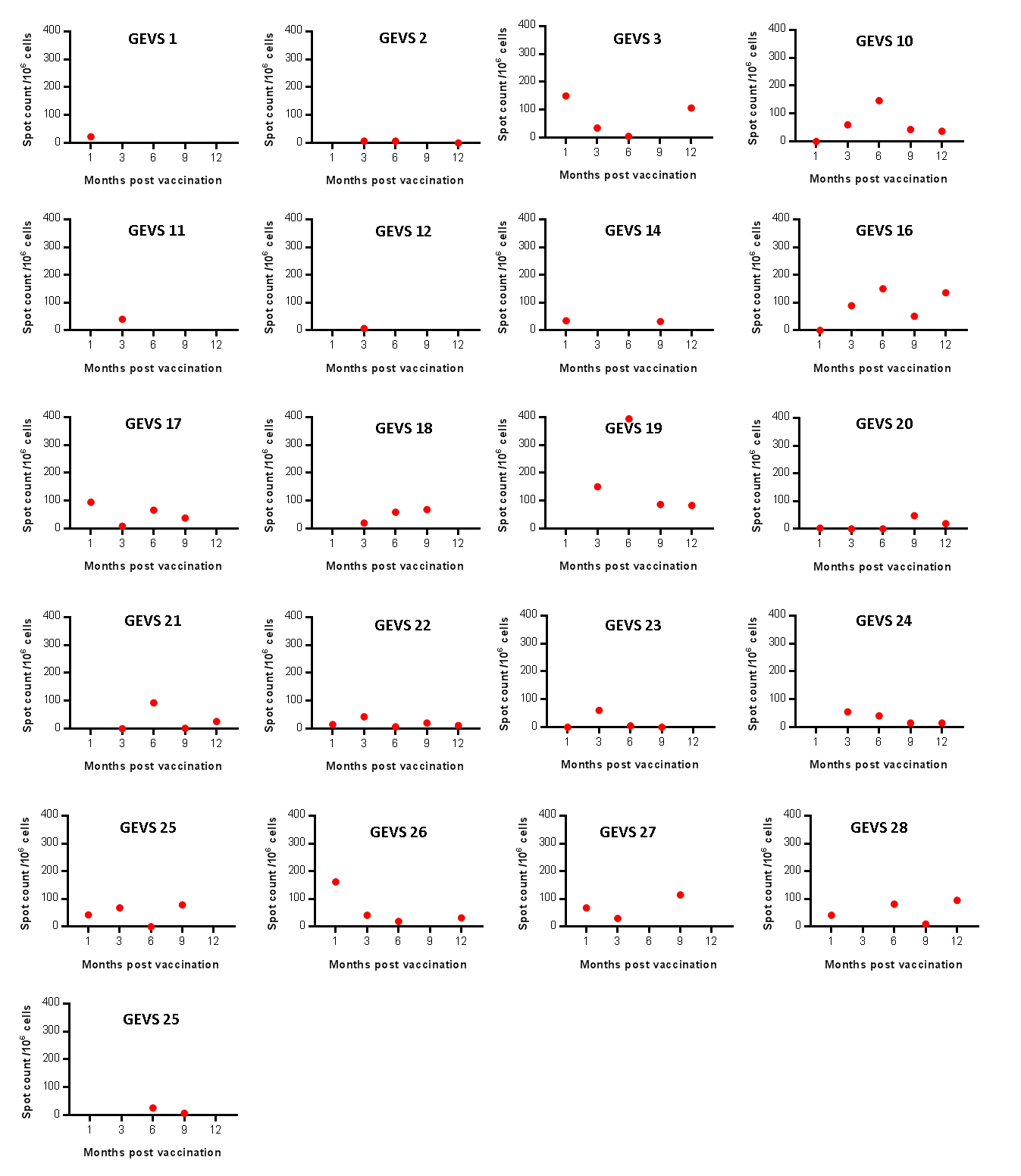
*

1. <https://niaid.github.io/spice/> [↑](#footnote-ref-1)
2. Roederer M, Nozzi JL, Nason MC. SPICE: exploration and analysis of

   post-cytometric complex multivariate datasets. Cytometry A. 2011

   Feb;79(2):167-74 [↑](#footnote-ref-2)
