# Supplementary material for: Postexposure Prophylaxis With rVSV-ZEBOV Following Exposure to a Patient With Ebola Virus Disease Relapse in the United Kingdom: An Operational, Safety, and Immunogenicity Report
Source: Clin Infect Dis. 2019 Nov 30;71(11):2872–9. doi: 10.1093/cid/ciz1165 (PMC7778350; doi:10.1093/cid/ciz1165)
Supplement: ciz1165_suppl_Supplementary_Forms [file ciz1165_suppl_supplementary_forms.docx]

**PATIENT INFORMATION SHEET AND CONSENT FORM**

**Glasgow Ebola Vaccine Follow-Up Study**

**Version 1 – October 2015**

This patient information leaflet is being provided to you as you have been identified as a person who was offered the rVSV-ZEBOV Ebola vaccine manufactured by NewLink/Merck. We now wish to follow-up your response to vaccination as part of a clinical study. We wish to find out if you have developed immunity to the infection and if you develop side effects following vaccination.

**Why was I offered VSV-GP (rVSV-ZEBOV) vaccination?**

You were identified as having had close contact with a patient recently diagnosed with Ebola virus infection. While the risk that you will develop infection yourself is considered to be extremely low, the use of this vaccine may have lowered the risk even further. As previously discussed with you, this vaccine is still being tested in clinical trials and is not yet licensed for use. This means that we cannot yet be sure how much this vaccine reduces the risk of developing infection.

**What was the vaccine that I was offered?**

The vaccine is a live vaccine consisting of a virus called vesicular stomatitis virus (VSV) that has been modified to contain a gene that comes from the Ebola virus. You cannot get Ebola virus from this vaccine as only a part of the virus is used in the vaccine. However, as this is a live vaccine, you may develop symptoms of a mild infection such as fever, headache, rash, muscle pain and aching joints.

**Can everyone be given this vaccine?**

As this vaccine is still an experimental medicine, we only offered it to people who had direct contact with the patient or with body fluids from the patient.

As the vaccine is a live vaccine, we did not offer it to people who had a weakened immune system, to pregnant or lactating women or to children under the age of 6 years.

**Has the vaccine been used in other people who have been exposed to Ebola virus?**

This vaccine was tested in over 7000 people during the recent outbreak of Ebola virus infection in Guinea. The study measured the number of cases of Ebola infection in people who had been given the vaccine either immediately after they had been in close contact with someone who developed Ebola virus infection (for example people living in the same household) or 21 days after they had been in contact with an affected patient. The study found that the people who had immediate access to the vaccine had a lower risk of developing Ebola virus infection than those who received vaccine after 21 days.

**How will you measure if I have any immunity to Ebola virus infection?**

We will measure the level of antibodies in your blood by taking blood samples at 7, 14, 28 days and 3, 6, 9 and 12 months after vaccination. We will also examine the vaccine rVSV-EBOV virus present in your blood to see how it changes over time as your immune response develops.

**What side effects might I develop after having the vaccine?**

The side effects of the vaccine that have been reported include fever, headache, rash and joint pains. As many as 1 in 2 people given the vaccine will develop fever. While we expect that this is likely to be a safe medicine, we do not yet know if there may be other side effects that may affect you in the future. We now wish to follow you up in the outpatient clinic to see if you develop any side effects following vaccination.

**How will you follow me up and how often will you see me in clinic?**

We will ask you to record your temperature every day for 21 days and we plan to follow you up in clinic at 7, 14, 28 days and 3, 6, 9 and 12 months post-vaccination.

**Will you carry out any other tests when you see me in clinic?**

We will obtain 36 ml of blood (4 x 9ml vials) from you at each clinic visit. We will store this blood to allow us to examine the response to vaccination at a later date. When you visit the clinic we will also obtain a sample of your saliva and urine in order to look for evidence of the vaccine virus in these fluids. If you wish, we will also test your seminal fluid for the presence of vaccine virus. If you develop a swollen joint that requires aspiration of fluid or have sampling of other body fluids carried out as part of your routine care, we may send a sample of this fluid in order to test for the presence of the rVSV-EBOV vaccine.

**What tests will be carried out on my blood and other body fluids?**

We will look for evidence of immunity to Ebola virus infection by measuring whether or not you have developed antibodies to Ebola virus and whether or not these are able to neutralise (kill) the virus. We will also examine the vaccine virus in your body fluids using a technique called deep sequencing. It is possible that some people develop more side effects than others because of their own genetic make-up. We will also retain your DNA in order to identify whether or not you have a predisposition to developing such side effects.

**Will the information gathered in this study be confidential?**

We will inform your GP that you have elected to take part in this study. We will also publish the results of the study in the scientific literature. However, we will not identify you personally and we will ensure that your participation in the study is entirely confidential.

**Scientific explanation of the rVSV study**

This recombinant, replication-competent vesicular stomatitis virus-based vaccine expressing a surface glycoprotein of Zaire Ebolavirus (rVSV-ZEBOV) is a promising Ebola vaccine candidate. The Lancet recently published a report of the results of an interim analysis of a trial of rVSV-ZEBOV in Guinea, West Africa undertaken by the World Health Organisation.

In this open-label, cluster-randomised ring vaccination trial, suspected cases of Ebola virus disease in Basse-Guinée (Guinea, West Africa) were independently ascertained by Ebola response teams as part of a national surveillance system. After laboratory confirmation of a new case, clusters of all contacts and contacts of contacts were defined and randomly allocated 1:1 to immediate vaccination or delayed (21 days later) vaccination with rVSV-ZEBOV (one dose of 2×10⁷ plaque-forming units, administered intramuscularly in the deltoid muscle). Adults (age ≥18 years) who were not pregnant or breastfeeding were eligible for vaccination. Block randomisation was used, with randomly varying blocks, stratified by location (urban vs rural) and size of rings (≤20 vs >20 individuals). The study was open label and masking of participants and field teams to the time of vaccination was not possible, but Ebola response teams and laboratory workers were unaware of allocation to immediate or delayed vaccination. Taking into account the incubation period of the virus of about 10 days, the prespecified primary outcome was laboratory-confirmed Ebola virus disease with onset of symptoms at least 10 days after randomisation. The primary analysis was per protocol and compared the incidence of Ebola virus disease in eligible and vaccinated individuals in immediate vaccination clusters with the incidence in eligible individuals in delayed vaccination clusters.

Between April 1, 2015, and July 20, 2015, 90 clusters, with a total population of 7651 people were included in the planned interim analysis. 48 of these clusters (4123 people) were randomly assigned to immediate vaccination with rVSV-ZEBOV, and 42 clusters (3528 people) were randomly assigned to delayed vaccination with rVSV-ZEBOV. In the immediate vaccination group, there were no cases of Ebola virus disease with symptom onset at least 10 days after randomisation, whereas in the delayed vaccination group there were 16 cases of Ebola virus disease from seven clusters, showing a vaccine efficacy of 100% (95% CI 74·7–100·0; p=0·0036). No new cases of Ebola virus disease were diagnosed in vaccinees from the immediate or delayed groups from 6 days post-vaccination. At the cluster level, with the inclusion of all eligible adults, vaccine effectiveness was 75·1% (95% CI –7·1 to 94·2; p=0·1791), and 76·3% (95% CI –15·5 to 95·1; p=0·3351) with the inclusion of everyone (eligible or not eligible for vaccination). 43 serious adverse events were reported; one serious adverse event was judged to be causally related to vaccination (a febrile episode in a vaccinated participant, which resolved without sequelae). Assessment of serious adverse events is ongoing.

The results of this interim analysis indicate that rVSV-ZEBOV might be highly efficacious and safe in preventing Ebola virus disease, and is most likely effective at the population level when delivered during an Ebola virus disease outbreak via a ring vaccination strategy.

The study was conducted by the WHO, with support from the Wellcome Trust (UK); Médecins Sans Frontières; the Norwegian Ministry of Foreign Affairs through the Research Council of Norway; and the Canadian Government through the Public Health Agency of Canada, Canadian Institutes of Health Research, International Development Research Centre, and Department of Foreign Affairs, Trade and Development.

**CONSENT FORM**

**VSV-GP (rVSV-ZEBOV) vaccination**

**Version 1 - October 2015**

**Please initial boxes**

1. I confirm that I have read and understood the information sheet dated October 2015 (version 1) and have had the opportunity to ask questions.

2. I understand that I do not have to participate in the study

3. I understand that sections of any of my medical notes may be looked at by responsible individuals from the hospital or from regulatory authorities. I give permission for these individuals to have access to my records.

4. I note that my GP will be informed that I am participating in this study

5. I agree to be vaccinated with VSV-GP (rVSV-ZEBOV) vaccine.

6. I agree to have blood, urine and saliva testing carried out as detailed in the patient information form including DNA testing

7. I agree to have seminal fluid testing carried out in the patient information form Y/N/NA

8. If I require to have other body fluids such as joint fluid sampled for routine clinical care, I agree for this to be tested later for the presence of the rVSV vaccine virus

---------------------------------- ------------------------------ ----------------------

Name of patient Signature Date

---------------------------------- ------------------------------ ----------------------

Name of person taking consent Signature Date
