# Supplementary material for: Postexposure Prophylaxis With rVSV-ZEBOV Following Exposure to a Patient With Ebola Virus Disease Relapse in the United Kingdom: An Operational, Safety, and Immunogenicity Report
Source: Clin Infect Dis. 2019 Nov 30;71(11):2872–9. doi: 10.1093/cid/ciz1165 (PMC7778350; doi:10.1093/cid/ciz1165)
Supplement: ciz1165_suppl_Supplementary_Questionaire [file ciz1165_suppl_supplementary_questionaire.docx]

# rVSV-EBOV vaccination follow-up protocol

## Follow-up time

Day 3 Day 10 Day 14 day 28

3 months 6 months 9 months 12 months

Has follow-up been carried out? Y/N

If not, why not?

Temperature

If temperature >38.5^o^C what action has been taken?

## Adverse events

New adverse event? Y/N

### Pain at injection site? Y/N

Absence of pain / mild pain with pressure / significant pain with pressure / significant pain while moving limb (interfering with normal activities)

Date pain started

Date ended

Description

### Induration

Absent <5mm 5-20mm 20-50mm >50mm

Date induration started

Date ended

### Myalgia

Absent Present without interfering with daily activities

Discomfort that interferes with daily activities but goes away with symptomatic treatment

Considerable interference with daily activities

Date myalgia started

Date ended

Description

### Fatigue

Absent Present but not interfering with daily activities Interfering to a certain extent with daily activities Considerable interference with daily activities

Date fatigue started

Date ended

Description

### Vomiting

Absent Present but not interfering with daily activities Interfering to a certain extent with daily activities Considerable interference with daily activities

Date vomiting started

Date ended

Description

### Diarrhoea

Absent Present but not interfering with daily activities Interfering to a certain extent with daily activities Considerable interference with daily activities

Date diarrhoea started

Date ended

### Headache

Absent Present but not interfering with daily activities Interfering to a certain extent with daily activities Considerable interference with daily activities

Date headache started

Date ended

Description

### Arthralgia

Absent Present but not interfering with daily activities Interfering to a certain extent with daily activities Considerable interference with daily activities

Date arthralgia started

Date ended

Which joints affected?

Description

### Myalgia

Absent Present but not interfering with daily activities Interfering to a certain extent with daily activities Considerable interference with daily activities

Date myalgia started

Date ended

Description

### Other side effects?

Date started

Date ended

Description

### Major adverse event

Death of subject

Life-threatening event

Serious adverse event causing incapacitation
